# Acute Oxidative Effect and Muscle Damage after a Maximum 4 Min Test in High Performance Athletes

**DOI:** 10.1371/journal.pone.0153709

**Published:** 2016-04-25

**Authors:** Heros Ribeiro Ferreira, Pamela Gill Ferreira, João Paulo Loures, José Fernandes Filho, Luiz Cláudio Fernandes, Hudson Sousa Buck, Wagner Ricardo Montor

**Affiliations:** 1 Santa Casa de São Paulo School of Medical Sciences, São Paulo, SP, Brazil; 2 Federal University of Parana, Curitiba, PR, Brazil; 3 University of São Paulo, São Paulo, SP, Brazil; 4 Federal University of Rio de Janeiro, Rio de Janeiro, RJ, Brazil; Universidad Pablo de Olavide, Centro Andaluz de Biología del Desarrollo-CSIC, SPAIN

## Abstract

The purpose of this investigation was to determine lipid peroxidation markers, physiological stress and muscle damage in elite kayakers in response to a maximum 4-min kayak ergometer test (KE test), and possible correlations with individual 1000m kayaking performances. The sample consisted of twenty-three adult male and nine adult female elite kayakers, with more than three years’ experience in international events, who voluntarily took part in this study. The subjects performed a 10-min warm-up, followed by a 2-min passive interval, before starting the test itself, which consisted of a maximum 4-min work paddling on an ergometer; right after the end of the test, an 8 ml blood sample was collected for analysis. 72 hours after the test, all athletes took part in an official race, when then it was possible to check their performance in the on site K1 1000m test (P1000m). The results showed that all lipoproteins and hematological parameters tested presented a significant difference (p≤0.05) after exercise for both genders. In addition, parameters related to muscle damage such as lactate dehydrogenase (LDH) and creatine kinase (CK) presented significant differences after stress. Uric acid presented an inverse correlation with the performance (r = -0.76), while CK presented a positive correlation (r = 0.46) with it. Based on these results, it was possible to verify muscle damage and the level of oxidative stress caused by indoor training with specific ergometers for speed kayaking, highlighting the importance of analyzing and getting to know the physiological responses to this type of training, in order to provide information to coaches and optimize athletic performance.

## Introduction

Olympic kayaking involves distances of 200, 500, and 1000 meters, thus, variables such as aerobic resistance, anaerobic power and strength are often included in the athletes training programs, in addition to all technical work on and off the water during training sessions [1–3].

Changes to volume and intensity during training accompanied by metabolic and hormonal changes have been demonstrated [4–8]. For kayakers, laboratory procedures have been used as alternative and reliable tools for training and evaluation [9], as training in the water points out to a series of limitations in terms of quantification, due to the standardization of natural variables that interfere in their practice, such as temperature, speed and wind direction [10].

As in rowing, kayak athletes also train indoors, when the training conditions in open water are not favorable and they can use ergometers, which have some advantages such as controlling the strength and the cadence in rowing [11]. So, the use of ergometers becomes a very interesting tool in the conditioning training, especially in the base period [12], besides being used in performance prediction reviews [9, 11, 13]. Therefore, understanding the biochemical laboratory responses for training and evaluating high performance sprint kayaking athletes has been seen as a performance improvement opportunity by many trainers and sport researchers [14, 15].

In some studies [9, 11, 16] with high-level kayakers, a correlation (r = 0.86) has been shown between the maximum work in a 4-min kayak-ergometer test (KE test) and the same time on the Olympic lane, suggesting that the KE test could be used as a performance predictor, due to the similarity. Studies [3, 11, 17–20] have also demonstrated that the 1000 meters kayak race conduct to high levels of oxidative stress and muscle damage.

The purpose of this study was to determine the levels of lipid peroxidation, muscle damage and physiological stress in elite kayakers after a maximum 4-min kayak-ergometer test (KE test) and possible correlations with performance on official races (P1000m).

## Materials and Methods

### Subjects

The sample consisted of thirty-two adult volunteer athletes—twenty-three male and nine female elite kayakers, with over three years’ experience in international events. All subjects were instructed not to perform intense exercises one day prior to evaluation. All participants were classified as healthy, non-smokers and did not make use of drugs or steroids in the season. They were also informed not to change their regular diet, and to avoid beverages which contained alcohol or caffeine for at least 24 hours prior to the tests.

### Ethics Statement

All subjects are professional athletes, so they are under constant training and performance evaluation and they were informed about the blood collections necessary for the study, signed a term of informed volunteer participation, which was approved by the Ethics Committee for Research in Human Beings from Santa Casa de São Paulo School of Medical Sciences, under the number 518.993, from the 29^th^ Jan 2014.

### Experimental design

The athletes were familiarized with the kayak-ergometer test (KE test) procedures. First of all, they performed a 10-min warm up, followed by a 2-min passive interval prior to starting the test. After 72 hours of the kayak-ergometer test, all athletes took part in an official race, when then it was possible to check their performance in the on site K1 1000m test (P1000m).

### 4-min kayak-ergometer test

The aim of the 4-min KE test is getting the athlete to perform a maximum muscle power throughout the test, without race strategies. The 4-min KE test consists of a 5-min free joint warm up, plus a 5-min warm up on a kayak-ergometer with 40W load, followed by a2-min passive interval. Immediately after the rest, the 4-min KE test begins.

### Biochemical analysis

Blood samples (~8 ml each sample) were obtained by using a regular syringe and needle, inserted into the ante-cubital vein; blood was transferred into a tube with EDTA for later analysis [3, 21].

Regents and instruments from Biosystems S.A were used to perform the enzymatic method for the quantitative determination of the serum lipoproteins in the samples, by using the spectrophotometric method. The VLDL cholesterol was calculated by triglyceride values, LDL was determined by the UV optimized kinetic method (DGKC) using kits from Wiener Labs, according to the protocol suggested by the same company. Hemoglobin was determined through the modified cyanmethemoglobin method with Autoblank (Sistem Cell-dyn1400). Uric acid was determined by using the enzymatic method, following the instructions of the Quimiuric kit. The CK levels were also measured by using the UV optimized kinetic method (DGKC) from UniTest and kits of the Wiener Labs. The UV optimized kinetic method (DGKC) was also used for determining the serum lactate dehydrogenase, following the Wiener Labs kit protocol.

### Statistics

Values are presented as mean & standard deviation. The ANOVA test was used to analyze possible differences between the variables pre and post tests, and the Pearson correlation test was used for checking possible variable correlational associations with performance. The dispersion of the values among pre and post test collections is presented in the form of percentage.

## Results and Discussion

The anthropometric characteristics, VO_2_Peak values in the 4-min test and performance in 1000m are presented in Table 1, and the values of energy and nutrients ingested are presented in Table 2. The KE test led to some changes in lipoproteins and hematological parameters, presented in Tables 3 and 4; a significant difference was found (*p*<0.05) in all parameters observed for both genders and the variation was presented in percentage (%).

**Table 1:** Anthropometric parameters, VO_2_Peak after the 4-min test and performance in the 1000m race.

	Male kayakers (n = 23)	Female kayakers (n = 9)
Age (years)	20.52 ± 4.47	20.11 ± 5.35
Height (cm)	177.09 ± 6.40	166.56 ± 7.38
Weight (kg)	73.70 ± 8.16	63.11 ± 6.64
VO_2_Peak (L.min^-1^)	2.73± .66	1.91± .65
VO_2_ Peak (mL.kg.min^-1^)	37.69± 8.25	31.16± 9.59
Performance 1000m (s)	250.93± 17.67	255.35± 7.25

**Table 2:** Energy and nutrients ingested by kayakers of both genders.

	Male kayakers (n = 23)	Female kayakers (n = 9)
Basal Metabolism (kcal)	1831.30± 229.90	1482.56± 279.30
Energy intake (kcal)	3020.52±701.34	1995.44±637.12
Proteins (g)	142.77±36.95	92.74±30.28
Proteins (% of energy)	19.53±5.77	19.12±6.26
Carbohydrates (g)	60.82±6.88	307.53±114.64
Carbohydrates (% of energy)	62.23±6.37	60.68±6.88
Fiber (g)	30.84±13.16	23.72±10.94
Fat (g)	65.62±25.92	46.09±15.69
Fat (% of energy)	8.57±2.25	9.32±1.17
Satured fat (g)	18.87±8.81	12.16±6.52
Monounsatured fat(g)	19.40±10.72	12.36±8.18
Polyunsaturated fat (g)	12.99±7.03	7.55±3.38
Cholesterol (mg)	307.74±152.56	172.13±72.69
Vitamin C (mg)	734.43±401.71	475.38±278.82
Vitamin A (RE μg)	1109.27±1488.26	666.23±458.99
Zinc (mg)	14.24±4.53	11.49±5.51
Copper (mg)	2.03±.52	1.40±.64
Iron (mg)	19.28±4.12	14.28±5.64
Magnesium (mg)	522.24±139.83	331.02±193.58
Manganese (mg)	3.50±1.64	2.64±1.68

**Table 3:** Lipoproteins and hematological parameters before and after the KE test for male kayakers.

	Before exercise	After exercise	Variance (%)
Total cholesterol (mg/dL)	154.70± 32.23	179.90± 37.51*†	+16,28
LDL cholesterol (mg/dL)	84.97± 28.08	93.28± 28.84*	+9,77
HDL cholesterol (mg/dL)	54.91±13.71	64.96± 15.08*	+18,30
Triglycerides (mg/dL)	74.09±35.66	125.78±62.90*†	+69,76
Hemoglobin (mg/dL)	16.99±6.37	18.01±7.66*	+6,00
Hematocrit (L/L)	46.85± 2.33	51.17± 8.25*	+9,22
Male kayakers (n = 23)			

* Significant difference comparing before and after exercise.

† Significant difference between male and female subjects. (p<0.05)

**Table 4:** Lipoproteins and hematological parameters before and after the KE test for female kayakers.

	Before exercise	After exercise	Variance (%)
Total cholesterol (mg/dL)	147.33±31.85	171.67±37.99*	+16,52
LDL cholesterol (mg/dL)	72.98±16.12	78.88±18.42*	+8,08
HDL cholesterol (mg/dL)	59.89±17.08	74.44±23.17*	+24,29
Triglycerides (mg/dL)	72.33±37.59	91.89±36.03*†	+27,04
Hemoglobin (mg/dL)	13.16± .78	14.27± .76*†	+8,43
Hematocrit (L/L)	39.68± 2.59	43.46± 1.78*†	+9,52
Female kayakers (n = 9)			

* Significant difference comparing before and after exercise.

† Significant difference between male and female subjects. (p<0.05)

The CK and LDH, which are connected to the markers of inflammation and stress, presented a significant difference after the KE test, while uric acid and cortisol presented no significant differences after exercise (Tables 5 and 6). Only CK after exercise presented low correlation with the P1000m (r = 0.42) for male kayakers, while for females only uric acid correlated with the P1000m (r = -0.76).

**Table 5:** Parameters of physiological stress and markers of inflammation before and after the KE test for male kayakers.

	Before exercise	After exercise	Variance (%)
Uric acid (mg/dL)	5.25± 1.17	5.12±.99	-2,47
Creatine Kinase (U/L)	712.35±739.91	861.83±749.78*†	+20,98
Cortisol (μg/dL)	16.19± 2.92	15.91± 4.39	-1,72
LDH	574.39± 110.23	648.87± 91.65*	+12,96
Male kayakers (n = 23)			

* Significant difference comparing before and after exercise.

† significant difference between male and female subjects. (p<0.05)

**Table 6:** Parameters of physiological stress and markers of inflammation before and after the KE test for female kayakers.

	Before exercise	After exercise	Variance (%)
Uric acid (mg/dL)	3.98±.65†	4.21±.81†	+5,77
Creatine Kinase (U/L)	227.89±107.61†	268.56±119.78*†	+17,84
Cortisol (μg/dL)	18.11± 4.67	18.50± 4.94	+2,15
LDH	500.89± 94.82	565.56± 92.68*	+12,91
Female kayakers (n = 9)			

* Significant difference comparing before and after exercise.

† Significant difference between male and female subjects. (p<0.05)

The main findings of the study were the alterations in lipoproteins and hematological parameters in addition to the CK and LDH responses after acute exercise, for both genders. There was a significant increase for all of them.

Studies found in the literature analyzed triglycerides and LDL responses after exercise in healthy subjects, and showed a drop in the concentration of triglycerides, while LDL levels remained unchanged [22], the opposite of what we found in the present study, where a significant increase for both parameters could be noticed; only the HDL presented a similar result with a significant increase after exercise in the male group [23, 24].

Also, other authors state that lipoproteins of kayakers remained unchanged after a K1 1000m effort and there are no differences between men and women, once again opposite to the findings of the present study, where significant differences were found in parameters related to lipoproteins [20, 25], moreover, a significant difference was also observed between the genders for triglycerides. In the literature, other authors have pointed out that levels of carotenoids are primarily responsible for the maintenance of LDL levels before and after exercise, since this is the main transporter [20, 26, 27]. Still, in the literature [26] we find a significant increase in triglyceride levels after exercise, and state that this response is a cellular defense to the negative effects of oxidative stress.

Uric acid, seen as an important antioxidant which acts directly on the reactive oxygen species [20], is used as a discrete marker of injury; other authors [28] show that after an average duration or high intensity exercise, a significant uric acid difference is noted from baseline in relation to the completion of the race. The values found in this study corroborate those found in the study of Teixeira and collaborators (2013) [20]. On the other hand, some authors [11, 15, 29–32] found significant differences after a K1 1000m test, after intense exercise of long duration, while in the present study no differences were found after 4-min of exercise in the KE test; the only difference was found between males and females, confirming the findings of Teixeira and collaborators (2013) [20].

For the CK variable, considered to be a discrete marker of injury, the results of the present study corroborate the studies of Teixeira and collaborators (2013), who observed alterations in the male group (pre and post exercise) and between genders. Other authors [8, 33–35] found alterations in the concentration of CK after a maximum effort in the 100m swimming freestyle in male and female subjects. One possible explanation for the results found in this study is the lipid peroxidation, which causes an increase in membrane permeability, facilitating the CK flow [20, 25].

Cortisol responses after exercise have also presented contradictory results. The values for cortisol were higher than those found in other studies [25, 36], although no significant differences were found. According to Schröder and collaborators (2001) [37], the cortisol response is dependent on both the intensity and duration of the exercise, and the model used was not sufficient to cause such alterations.

The LDH variable presented the same behavior as the CK, confirming the data found by other authors [38] which showed significant differences in LDH after a maximum test [39, 40].

When relating the parameters of physiological stress with performance, a positive correlation was observed for CK in the male group, and an inverse correlation for uric acid for the female group (r = .42; r = -.76 respectively). Some authors [41, 42] reported that performance responses couldn’t be associated with alterations in biomarkers in different periods of training, and other authors [43], despite correlations between biomarkers of lipid peroxidation and performance not being found, showed that during the tapering period, when the training loads are reduced, the same occurs with biomarkers, which is considered to be an important performance factor.

## Conclusion

This study indicates that the KE 4min test for elite kayakers induces oxidative stress and muscle damage for both genders (male and female), reinforcing the importance of body stress during indoors training with an ergometer, which can be used, instead of the water outdoors training. We also show a laboratory assessment tool, as a means of training and controlling tactical performance and individual technique. We recommend that further studies are performed in order to compare the performances of kayak athletes with ergometers, as well as the inclusion of more biochemical variables.

## Abbreviations

KE: kayak ergometer
K1: kayak individual
LDH: lactate dehydrogenase
CK: creatine kinase
P1000m: Performance on 1.000m
W: Watts
EDTA: Ethylenediaminetetraacetic acid
VLDL: very low density lipoprotein
HDL: high density lipoprotein
LDL: low density lipoprotein
DGKC: German Society of Clinical Chemistry
UV: ultraviolet
CK: Creatine kinase
CK MM: muscle isoform of creatine kinase
VO_2_Peak: peak oxygen uptake
